# Impact, distress and HRQoL among Malaysian men and women with a mobility impairment

**DOI:** 10.1186/1477-7525-4-95

**Published:** 2006-12-12

**Authors:** RoseAnne Misajon, Lenore Manderson, Julie F Pallant, Zaliha Omar, Elizabeth Bennett, Rameezan Begam Abdul Rahim

**Affiliations:** 1 School of Psychology, Psychiatry & Psychological Medicine, Monash University, 900 Dandenong Rd, Caulfield East, Victoria 3145, Australia; 2 Faculty of Life and Social Sciences, Swinburne University of Technology, P.O. Box 218, Hawthorn, Victoria 3122, Australia; 3 Department of Allied Health Sciences, Faculty of Medicine, University of Malaya, Kuala Lumpur, Malaysia; 4 School of Population Health, The University of Melbourne, Victoria 3010, Australia

## Abstract

**Background:**

Although non-communicable and chronic disease now accounts for 47% of the global burden of disease, little is known of the everyday experiences and social aspects of disability and disablement in middle and low income countries. This article aims to address this gap by exploring the subjective experience of mobility impairment in Malaysia. Specifically, it examines health-related quality of life and the impact and distress related to impaired mobility, and investigates any gender differences in relation to the experience of disability.

**Methods:**

The data were collected as part of an interdisciplinary, multi-country study known as RESILIENCE (Research into Social Inclusion, Locomotive Impairment and Empowerment through Networking, Collaboration and Education). Cluster sampling was used to administer the EQ-5D and the Perceived Impact of Problems Profile (PIPP) to 210 adults from Selangor state, west coast Peninsular Malaysia.

**Results:**

The participants consisted of 94 males and 116 females, aged between 18–90 years (mean 60 years), with the majority being Malay. The majority of participants were also married, from rural areas and had primary education only. Very few participants lived alone. In addition, males were more likely to attribute their impaired mobility to an accident. The majority of participants with mobility impairment experienced a moderate to high level of pain/discomfort (79%) and anxiety/depression (72%), and at least some problems with performing usual activities (71%), as measured by the EQ-5D. In addition, using the Perceived Impact of Problems Profile (PIPP), participants also reported high levels of impact and distress related to participation in community life. In general, males reported higher impact and distress across several items, most significantly in regard to participation in community activities, moving around the neighbourhood, ability to live independently, and ability to assist their family members.

**Conclusion:**

This paper provides preliminary data regarding the health-related quality of life among Malaysians with impaired mobility, and highlights the multifaceted impact of disability and the importance of acknowledging the diverse cultural contexts in which disability can occur. It also raises questions regarding gender differences in the subjective experience of disability in Malaysia.

## Background

Non-communicable and chronic disease currently account for 60% of deaths globally per annum and 47% of the global burden of disease [1], yet there has been little research interest in countries outside Western Europe and North America [2]. Furthermore, the emphasis in research worldwide until recently has focused on etiology, treatment and epidemiology, with limited attention to non-clinical and especially social aspects of these conditions [2]. So although the increasing prevalence of chronic disease is not confined to advanced economies, we know far less of the everyday experiences and social aspects of disability and disablement in middle and low income countries [but see [3,4]]. Our own research, on which we draw for this article, has been concerned with personal and social environmental factors that contribute to disability. In assessing these, as described below, we developed instrumentation to explore the perceived impact and distress of people with mobility impairment [5].

Musculoskeletal conditions, including those of acute onset (e.g. injury, trauma), and short or long-term disorders (e.g. multiple sclerosis, arthritis), are the most common cause of physical disability and severe chronic pain. The frequency of such conditions increases with age, and with longer life expectancies globally, there will be a major impact on societies. The impact of locomotor limitation on quality of life is multi-dimensional with significant limitations in activity and restrictions in participation. For instance, locomotor impairment can make social participation difficult, place limitations on lifestyle and ability to perform self-care tasks, increase dependency on others, reduce income and employment potential, and overall, reduce quality of life [6,7].

In this article, we examine health-related quality of life, impact, and distress among people living with mobility limitations in Peninsular Malaysia. Malaysia is a middle income country, with an ethnically diverse population estimated to be around 25.5.million (in 2005). Today, some 62% are Malay and other indigenous, 28% Chinese, and 10% Indian. Life expectancy is estimated at 72 years (70 male, 75 female); by 2020, 9.8% will be over the age of 60 and 6.2% over 65. Changes in patterns of employment, physical activity levels, diet and smoking, have all contributed to the increased incidence of chronic disease, such that the epidemiological profile and individual risk factors of disease in Malaysia are rapidly approaching those of high-income countries. In 1997 (most recent available representative community data), 29.9% of adults 30 years of age or older had possible or diagnosed hypertension; the overall prevalence of diabetes mellitus in the same age group was 8.3%; 6.9% of the population reported impairments, of whom some two thirds had physical impairments and 1.5% reported disability as a consequence of these impairments [8]. The majority of physical impairments derive from adult onset chronic and degenerative conditions and from injury, the latter more likely to affect men as well as those who are younger, have low education and low income [8].

Previous research exploring gender and disability indicates the disadvantageous and marginalised situation for women with disabilities, with researchers observing women with disabilities as often experiencing a 'double' disadvantage due to the combined effects of gender and disability. Findings suggest that women with a disability, as compared with men with a disability and women without a disability, faced a double disadvantage in terms of employment, education, and income levels, and were much less likely to marry, and more likely to become divorced if married prior to onset of the disability [9-11]. Malaysian women in general were found to have lower health-related quality of life than men, as measured by the SF-36 [12].

To ensure that policy and programs are informed by evidence, the personal and social experiences of individuals whose lives are limited by their health status requires documentation, yet little research has been conducted to date. This article addresses this gap by providing some preliminary data regarding the experience of limited mobility from the person's perspective. Specifically, it aims to examine health-related quality of life and the impact and distress related to mobility impairment, and to investigate the different experiences of disability for males and females living in Malaysia.

## Methods

The data were collected as part of an interdisciplinary, multi-country study known as RESILIENCE (REsearch into Social Inclusion, Locomotive Impairment and Empowerment through Networking, Collaboration and Education), conducted with people living with physical impairments in Australia and Southeast Asia, including Malaysia. The study aimed to explore the social experience and cultural perceptions of disability, consider how contextual and environmental factors affect individual functioning and disability, and describe the consequent disadvantages and marginalization of individuals and their families. Field research for the first phase of this study was conducted from late 2003 to early 2004 in Selangor state, west coast Peninsular Malaysia. Within this state, 54% of the total population is Malay, 26% Chinese, 4% Indian and 4% non-Malaysian citizens. Because of the lack of community-based studies on chronic health conditions and/or disability, we chose to include both quantitative and qualitative methods to collect baseline data. In this article, we focus primarily on the quantitative data.

A liberal criterion was used to identify potential participants, rather than identifying participants on the basis of medical diagnosis, we included any person who had condition that they or others considered effected their mobility, such as limb amputation from injury or disease, restricted mobility from accident (e.g. spinal cord injury), stroke, degenerative conditions such as arthritis, or foot or leg deformity. Any of these conditions might, we theorized, result in physical impairment that could restrict an individual's involvement in social and economic life, and so have potential effects on their well-being.

The study used a cluster sampling method based on that of the Expanded Program for Immunization the World Health Organization. This is a simplified cluster sampling method which allows the random selection of seven children in 30 clusters, or communities. The method is able to be used by teams of data collectors who lack skills in sampling, and where comprehensive sampling frames are not available. The method does not randomly select households, but instead requires the interviewer to follow a particular path through the community, selecting every seventh household. Although the method permits some bias, its validity is good, and it has been widely used by teams of researchers to collect data on a wide range of health and social issues [13,14].

We first randomly selected 30 clusters from a sampling frame of all administrative communities in Selangor State. Fifteen of these were urban communities and fifteen were rural villages. In each cluster, seven people aged 18 years or above who, for any reason, had difficulty in moving about, were selected by local staff from each selected health centre located in the main town of the selected districts. Hence, selection of the 210 participants was purposive rather than random. All people invited agreed to participate in the study.

Data were collected by academic staff working in medical rehabilitation and medical graduates enrolled in a master's degree in the field, all trained to administer the research instruments and to collect, manage, and analyse data. Instrumentation was developed in English and translated into Bahasa Malaysia, and although formal back translation was not undertaken, the intent and precise meanings of terms were discussed at length and pre-tested during the course of training. Where required by study participants, the questionnaire was administered in a Chinese dialect or in Tamil. The questionnaire completed by participants consisted of 3 main parts:

(1) Socio-demographic background: Details were collected on age, sex, education (in years), ethnicity, religious background, marital status and household size.

(2) Health status: Participants were asked about their mobility problems, duration and causes, and comorbidities. They were also required to respond to the EQ-5D generic measure of health status, developed by the EuroQol Group [15]. This instrument was chosen due to it being a short, standardized, validated non-disease-specific instrument, available in Malay (for Malaysia), Bahasa Indonesia, Thai, and Chinese (for Singapore) [16]. The EQ-5D requires respondents to indicate their own health on a choice of three levels according to self-care, mobility, usual activities, pain/discomfort, and anxiety/depression. A visual-analogue scale (VAS) was also used for participant self-rating of their general health on the day. For the purposes of this study, we are interested in the individual items and hence, will be reporting on the EQ-5D descriptive system for the five dimensions, not the EQ-5D index values. Also, to our knowledge, there is currently no available normative data for Malaysia.

(3) Perceived impact and associated distress related to mobility impairment: The Perceived Impact of Problem Profile (PIPP) was developed as a relatively short, self-report instrument to assess both the impact and the distress of health problems from the individual's perspective (see [5] for more details). The development of the 23 items in the PIPP was guided in part by The International Classification of Functioning, Disability and Health (ICF), introduced by the World Health Organization [17]. The key domains include Mobility, Self-care, Relationships, Participation and Psychological well-being. The instrument was designed to be generic to allow for comparisons across conditions, and aimed at measuring the impact and distress associated with a condition rather than the person's ability to perform a particular task. For each item, respondents were asked to rate on a 6-point scale (a) 'how much impact has your current health problems had on [item of function or activity]'; and (b) 'How much distress has been caused by the impact of your health problem on [same item of function or activity]'. The 6-point scale was anchored on either end by 'no impact' and 'extreme impact' for the Impact scale and by 'no distress' and 'extreme distress' for the Distress scale.

The quantitative data analyses were conducted using SPSS statistical software (version 14.0) to investigate gender differences in regard to socio-demographic characteristics, health profile, health-related quality of life, and the experiences of impact and distress on various personal and social domains as a result of health problems. Spearman's correlations were used to explore the relationship between impact and distress.

Additional qualitative interviews and small group discussions were conducted with 30 individuals: village headman and other community leaders, religious leaders, health service providers working at the health centres, individuals with impairments, and primary care-givers. Like the survey questionnaire, interviews were conducted in the language of choice of the interviewee, mostly in Malay, but also in English, a Chinese dialect or Tamil. Interviews and focus group discussions were tape-recorded and transcribed, and translated if recorded in a Chinese dialect or Tamil. All textual data were entered into computer and analyzed thematically. Where qualitative data are used to elaborate points in this paper, we attribute the comments identifying respondents by ethnicity and gender.

## Results

### Socio-demographic and health profile

The socio-demographic characteristics and health profile of the total sample of survey respondents, and separately for males and females, are presented in Table 1. Participants were between 18–90 years of age (mean 60 years). Females had a slightly higher age mean than males [t(208) = -2.46, p < .05]. To control for any age-related differences in health-related quality of life and impact and distress, age was used as a covariate in later analyses examining gender differences with these variables. The majority of participants had primary education only (1–6 years; 72%), although some had secondary (7–12 years; 23%); only a few had tertiary education (≥ 13 years; 5%). This is consistent with the age and areas of residence of the study population; 72% were from rural and 28% were from urban areas. Significant gender differences in education were found, with females reporting fewer formal years of education than males [t(208) = 4.33, p < .001]. In terms of marital status, the majority of participants were married. However, significant differences were found for gender [χ^2^(2) = 37.17, p < .001]. Three-quarters of males were married, whereas for females, almost half were married and half separated, divorced or widowed. This could be attributed to the higher life expectancy of women and the higher age mean of the women in this sample.

**Table 1:** Socio-demographic and health profile of participants (N = 210)

**Socio-demographic and health**		**Total (N = 210)**	**Males (N = 94)**	**Females (N = 116)**	**p value**
		**%**^**a**^	**%**^**a**^	**%**^**a**^	
**Gender**			44.8	55.2	
**Age**	*Range*	18–90	18–90	18–90	
	*Mean yrs & SD*	60.2 ± 16.6	**57.1 ± 17.5**	**62.7 ± 15.5**	.015
**Education level**	*Mean yrs & SD*^*1*^	5.1 ± 4.2	**6.4 ± 3.6**	**4.0 ± 4.3**	<.001
	Primary (1–6)	72.4	63.8	79.3	
	Secondary (7–12)	22.8	20.9	16.4	
	Tertiary (≥ 13)	4.8	5.3	4.3	
**Residency**	Rural	72.4	74.5	70.7	
	Urban	27.6	25.5	29.3	
**Marital status**	Never married	10.5	**16.0**	**6.0**	
	Married	60.0	**75.5**	**47.4**	<.001
	Separated/divorced/widow	29.5	**8.5**	**46.6**	
**Religion**	Muslim	71.9	64.9	77.6	
	Buddhist	9.5	8.5	10.3	
	Confucian	2.4	4.3	0.9	
	Christian	2.4	2.1	2.6	
	Hindu	12.9	18.1	8.6	
	None/Other	1.0	2.1	0.0	
**Ethnicity**	Malay	70.5	64.9	75.0	
	Chinese	13.8	13.8	13.8	
	Indian	15.2	20.2	11.2	
	Other	0.5	1.1	0.0	
**Adults in household**	no other adults	4.8	1.1	7.8	
	1 other adult	23.3	26.6	20.7	
	2 or more other adults	71.9	72.3	71.6	
**Child/ren in household**	no children	46.2	42.6	49.1	
	1 child	15.2	17.0	13.8	
	2 or more children	38.6	40.4	17.1	
**Duration – mobility problems**	*Range (years)*	0 – 60	0 – 44	0 – 60	
	*Mean years & SD*	6.8 ± 10.9	6.0 ± 9.2	7.5 ± 12.1	
**Cause of mobility problems**	Sickness/Illness	61.0	62.8	59.5	
	Accident	23.8	**64.0**	**15.5**	.002
	Since birth	2.4	3.2	1.7	
	Don't know	11.9	**5.3**	**17.2**	.009
	Others	9.0	4.3	12.9	
**Other health probs.**	Hypertension	43.8	38.7	47.8	
	Diabetes	35.4	34.0	36.5	
	Arthritis	29.0	**16.0**	**39.7**	<.001
	Stroke	18.2	21.3	15.7	
	Heart disease	14.8	13.8	15.5	
	Lung dis.(inc.asthma)	8.7	8.6	8.7	
	Tuberculosis	2.9	4.3	1.7	
	Others	6.2	4.4	7.0	

^a^unless otherwise stated

Consistent with official statistics of the ethnic composition of the state, the majority of participants were Malay (71%). Of the participants who identified themselves as being Chinese (13.8%) or Indian (15.2%), most (approx 90%) would have conducted their interview in Mandarin, Cantonese or Tamil, with only a small percent in Malay. Nearly three quarters of the sample were Muslim (all Malays and some others), followed by Hindu, Buddhist, Christian and others. No significant gender differences were found in relation to ethnicity or religion. In addition, there were no gender differences in regard to household composition. Very few people lived in a house with no other adult, with more than 70% living with 2 or more adults. Almost half of the sample lived in households where there were no children currently living there. Mean household size (total all adults and children including respondent) was 3.69.

The health profile of respondents is also listed in Table 1. The majority of participants indicated sickness or illness to be the cause of their immobility. However, a significantly higher percentage of males attributed their mobility impairment to an accident, while significantly more women did not know the cause of their mobility problem (both at p < .01). In terms of co-morbidities, the most common other health problems included hypertension, diabetes and arthritis, followed by stroke and heart disease. Significantly more women than men indicated having arthritis (p < .001), possibly reflecting age differences between males and females in this study, and the high proportion of males who reported accident-related mobility impairment.

### Health-related quality of life

The responses on the EQ5D, including differentiation by gender, are presented in Table 2. Consistent with selection criteria, the majority of the respondents (78%) reported experiencing some problems with mobility and a smaller proportion (15%) indicated confinement to bed. Only 8% felt they were able to walk about without problems. In regard to self-care, almost half of the participants indicated having problems, with 30% having some problems while 16% unable to wash or dress themselves.

**Table 2:** Percentage frequency distribution of the EQ5D descriptive system

	**Total**	**Males**	**Females**
**N**	210	94	116
**Mobility**			
- no problems	7.6	7.4	7.8
- some problems	77.6	75.5	79.3
- confined to bed	14.8	17.0	12.9
**Self-Care**			
- no problems	54.3	47.9	59.5
- some problems	29.5	30.9	28.4
- unable to	16.2	21.3	12.1
**Usual Activities**			
- no problems	29.5	29.8	29.3
- some problems	42.9	36.2	48.3
- unable to	27.6	34.0	22.4
**Pain/Discomfort**			
- none	21.0	25.5	17.2
- moderate	68.6	60.6	75.0
- extreme	10.5	13.8	7.8
**Anxiety/Depression**			
- none	28.1	28.7	27.6
- moderate	54.8	50.0	58.6
- extreme	17.1	21.3	13.8
**Mean EQ VAS score**			
Mean & SD	59.6 ± 21.7	56.7 ± 24.1	62.0 ± 19.3

Using age as a covariate, significant gender differences were found for Mean EQ VAS score [F(1) = 5.09, p = .025]

Only 30% of the participants reported having no problems with performing usual tasks, such as work, study, housework, family or leisure activities. In fact, almost 30% of the sample indicated being unable to perform usual tasks. Furthermore, almost 80% of participants reported having moderate or extreme pain and/or discomfort, while more than 70% reported moderate or extreme anxiety and/or depression. No gender differences were found in regard to the five domains using non-parametric analyses. However, gender differences were found for the Mean EQ VAS score, the participant's self-rating of their own health state on the day of the interview. Males reported having significantly worse health state than females (p < .05). Both means were just above the mid-point of the 100-point visual analogue scale.

### Impact and distress

The impact and distress related to impairment (as measured by PIPP) for the total sample, and for males and females separately, are reported in Table 3. The items for which participants' health problems had greatest impact were mainly from the participation and mobility domains. More specifically, their health had greatest impact on their *ability to work*, *participate in community activities*, *participate in activities enjoyed*, *ability to carry things*, *move around the neighbourhood*, and *overall satisfaction with life*. The items for which their impairment had least impact were mainly the self-care and relationship items, such as *ability to feed*, *dress*, and *wash self*, *have close relationships *or *relate to relatives, neighbours and friends*. The items for which participants reported least distress were also from the self-care and relationships domains. In contrast, the items which were linked to greatest distress were from the psychological well-being domain, namely *overall satisfaction with life*, as well as from the participation domain, such as *ability to participate in community activities, activities enjoyed*, and *ability to work*.

**Table 3:** Mean IMPACT and DISTRESS scores for the 23 PIPP items, and gender differences using age as a covariate

	**IMPACT**				**DISTRESS**			
	**Total N = 210**	**Males N = 94**	**Females N = 116**	**p value**	**Total N = 210**	**Males N = 94**	**Females N = 116**	**p value**
**Mobility**								
Sit or stand	4.0 *± 1.7*	4.1 *± 1.7*	4.0 *± 1.7*	.262	3.1 *± 1.7*	3.2 *± 1.8*	3.1 *± 1.5*	.483
Carry things	3.9 *± 1.8*	4.3 *± 1.7*	3.5 *± 1.7*	.034	2.9 *± 1.7*	3.1 *± 1.8*	2.8 *± 1.7*	.671
Use a vehicle	3.7 *± 1.9*	4.3 *± 1.8*	3.2 *± 1.8*	.042	2.9 *± 1.9*	3.4 *± 1.9*	2.5 *± 1.7*	.024
Move around own house	3.2 *± 1.9*	3.5 *± 1.9*	3.1 *± 1.8*	.005	2.6 *± 1.7*	2.7 *± 1.8*	2.5 *± 1.7*	.099
Move around neighborhood	3.8 *± 1.9*	4.2 *± 1.9*	3.5 *± 1.9*	.005	2.9 *± 1.9*	3.3 *± 1.9*	2.6 *± 1.8*	**.001**
								
**Self-Care**								
Wash self	2.4 *± 1.8*	2.7 *± 1.9*	2.1 *± 1.7*	**.001**	2.1 *± 1.6*	2.4 *± 1.8*	1.9 *± 1.4*	.018
Use the toilet	2.9 *± 2.0*	3.0 *± 2.1*	2.9 *± 1.9*	.021	2.5 *± 1.8*	2.7 *± 2.0*	2.3 *± 1.6*	.022
Dress self	2.3 *± 1.8*	2.5 *± 1.9*	2.0 *± 1.6*	.012	2.0 *± 1.6*	2.2 *± 1.8*	1.8 *± 1.3*	.017
Feed self	1.9 *± 1.6*	2.1 *± 1.8*	1.7 *± 1.4*	.093	1.7 *± 1.4*	1.9 *± 1.6*	1.5 *± 1.2*	.241
								
**Relationships**								
Relate to people in authority	3.0 *± 2.0*	3.3 *± 2.0*	2.8 *± 2.0*	.039	2.5 *± 1.8*	2.8 *± 1.8*	2.2 *± 1.7*	.087
Relate to neighbors and friends	2.8 *± 1.8*	3.0 *± 1.9*	2.6 *± 1.7*	.009	2.3 *± 1.6*	2.5 *± 1.7*	2.2 *± 1.5*	.048
Relate to relatives	2.7 *± 1.8*	2.9 *± 1.9*	2.6 *± 1.7*	.012	2.3 *± 1.6*	2.3 *± 1.7*	2.2 *± 1.6*	.013
Have close relationships	2.1 *± 1.7*	2.2 *± 1.8*	1.9 *± 1.5*	.080	2.0 *± 1.6*	2.2 *± 1.8*	2.7 *± 1.3*	.038
								
**Participation**								
Assist other family members	3.3 *± 1.9*	3.8 *± 2.0*	2.8 *± 1.8*	**<.001**	2.6 *± 1.8*	2.9 *± 2.0*	2.3 *± 1.5*	**.004**
Participate in family activities	2.4 *± 1.8*	2.6 *± 1.9*	2.2 *± 1.7*	.016	2.1 *± 1.6*	2.3 *± 1.8*	1.8 *± 1.4*	.025
Participate in community activities	4.0 *± 1.9*	4.2 *± 1.8*	3.9 *± 2.0*	.008	3.2 *± 1.9*	3.5 *± 2.0*	2.9 *± 1.8*	**.004**
Participate in activities you enjoy	4.0 *± 1.8*	4.4 *± 1.7*	3.7 *± 1.9*	.008	3.2 *± 1.8*	3.5 *± 1.8*	2.9 *± 1.8*	.013
Work	4.2 *± 2.0*	4.8 *± 1.7*	3.5 *± 2.0*	.006	3.1 *± 1.9*	3.8 *± 2.0*	2.3 *± 1.6*	.012
								
**Psychological well-being**								
Overall satisfaction with life	3.7 *± 1.6*	3.9 *± 1.7*	3.5 *± 1.5*	.010	3.2 *± 1.6*	3.5 *± 1.7*	2.9 *± 1.6*	**.002**
Moods and feelings	3.2 *± 1.6*	3.4 *± 1.6*	3.1 *± 1.5*	.093	3.0 *± 1.7*	3.3 *± 1.8*	2.7 *± 1.6*	.025
Sense of confidence	2.9 *± 1.7*	3.3 *± 1.8*	2.7 *± 1.6*	.013	2.7 *± 1.7*	3.0 *± 1.8*	2.4 *± 1.6*	.013
								
**Independence**								
Live independently	3.4 *± 2.0*	3.7 *± 2.0*	3.1 *± 2.0*	.004	3.0 *± 1.9*	3.3 *± 2.0*	2.6 *± 1.8*	**<.001**
Reliance on others	3.4 *± 1.9*	3.6 *± 1.8*	3.2 *± 1.9*	.017	2.9 *± 1.8*	3.2 *± 1.9*	2.6 *± 1.7*	.035

^a^Highlighted p values are significant after bonferonni adjustments

Another clear pattern emerging from the data is that males consistently reported higher impact and distress across all domains than females. Although a statistically significant difference was not found for every variable, males and females were significantly different for most items at least at p < .05 using age as a covariate. However, discussion of results will focus mainly on significant differences after Bonferonni type adjustments were made for inflated Type 1 error (p < .002). The most notable differences in terms of impact was that males reported significantly higher impact for *ability to wash self *and *ability to assist other family members *than females (refer to Table 3). There was no significant gender difference in terms of distress related to *ability to wash *self, but males did report significantly more distress than females for *ability to assist other family members *(refer to Table 3). Males also indicated significantly greater distress than females for *ability to move around neighbourhood*, *ability to participate in community activities*, *overall satisfaction with life*, and *ability to live independently*.

A general pattern for all items was that mean impact was higher than mean distress, indicating that an impact on a particular item did not necessarily equate to similar levels of distress. Analyses of the relationship between impact and the resulting distress for each item revealed that, as would be expected, all correlations were significant at p < .001 (refer to Table 4). For all items except *close relationships*, correlations were <.90 (range .61–.88). The correlation between impact and distress for *close relationships *was .95 indicating that while the participants reported having low impact and distress for this item, any impact on close relationships would likely result in a very similar level of distress. In terms of gender differences, the correlations between impact and distress for each item were similar when analysed separately for males and females (refer to Table 4). However, the correlation between impact and distress was higher for females by at least .10 for items *ability to carry things*, *ability to move around house*, and *participate in activities you enjoy*, and higher for males than females for *ability to participate in community activities*.

**Table 4:** Spearman's correlation between impact and distress for each item for total sample, and males and females separately

	**Total**	**Males**	**Females**
**N**	210	94	116
**Mobility**			
Sit or stand	.69	.69	.68
Carry things	.61	**.50**	**.70**
Use a vehicle	.67	.62	.66
Move around own house	.75	**.69**	**.81**
Move around neighborhood	72	.74	.71
			
**Self-Care**			
Wash self	.82	.81	.81
Use the toilet	.81	.84	.78
Dress self	.87	.88	.87
Feed self	.87	.90	.84
			
**Relationships**			
Relate to people in authority	.84	.88	.80
Relate to neighbors and friends	.78	.74	.80
Relate to relatives	.80	.79	.80
Have close relationships	**.95**	.98	.90
			
**Participation**			
Assist other family members	.71	.72	.69
Participate in family activities	.87	.84	.87
Participate in community activities	.70	**.76**	**.65**
Participate in activities you enjoy	.69	**.62**	**.74**
Work	.67	.66	.59
			
**Psychological well-being**			
Overall satisfaction with life	.76	.76	.73
Moods and feelings	.87	.88	.85
Sense of confidence	.84	.86	.81
			
**Independence**			
Live independently	.82	.82	.81
Reliance on others	.81	.81	.81

All correlations were significant at p < .001 (Bonferonni adjustment p < .002

## Discussion

### Living with impaired mobility in Malaysia

This study provides preliminary data on the experience of impaired mobility in Malaysia from the participant's perspective, and suggests areas in which more detailed exploration may be warranted. In terms of health-related quality of life, the findings indicate that the majority of people with mobility impairment experience at least some problems with performing usual activities, moderate to high levels of pain or discomfort and at least moderately high levels of anxiety or depression, reflecting the multifaceted impact of many of the health conditions experienced by the participants.

Self-care was less of an issue, as more than half indicated having no problems with self-care, although 16 percent did report being unable to perform self-care tasks. The results on the PIPP also indicated that impact and distress associated with self-care was relatively low in comparison with other domains. This may partly be explained by the ideals of family obligation and conventions of reciprocity between family members in Asian culture [18], whereby considerable social pressure exists for family members to fulfil their 'duty' to care for ailing family members. Therefore getting some assistance with self-care activities from family members may not be considered outside of the norm within families and hence, not register as having an impact or causing distress. In addition, very few participants in this study (less than 10%) lived alone, and it is likely that most lived with other family members who were able and willing to assist in self-care tasks.

Given family commitment to care, it is also then not surprising that individuals reported little impact on their ability to have a close relationship with another person (e.g. husband or wife) or their inclusion and participation in family activities. This may relate to mean age of participants, and results may have differed if more of our participants were younger and unmarried. Nonetheless, for those with established family networks, while a disability can place a profound burden on family resources, interpersonal relationships can prove to be resilient. The very high correlation (i.e. r = .95) between impact and distress for close relationships suggests that if participants were to register an impact on their close relationships, very similar distress levels would also be reported. For all other PIPP items, while the correlations between impact and distress were relatively high, all were below .89, confirming that measuring both impact and distress does not result in redundant information, and instead can highlight items which may be associated with high impact but lower distress, and/or vice versa.

Another domain, which the findings highlight as particularly important, was participation in community life and in activities enjoyed, similar to previous findings in other [e.g. [6]]. These items were rated as being quite high in terms of impact and distress. People indicated that their mobility impairment made it difficult to mix with neighbours at will, attend various community or other social events and festivities, or participate in ritual and religious observations. Despite considerable variation as a result of rapid social and economic changes, particularly in rural settings, high value is placed on community engagement, religious observation and interaction with one's neighbours, all of which play an integral part of daily living [19-22]. The physical environment, particularly in rural areas, would add to the difficulties already faced by many of our participants in terms of physically navigating themselves around the neighbourhood. Many of the study participants used assistive devices such as canes and, in a few cases, basic wheelchairs. Consistent with low education and occupation, however, few were able to afford high quality gait aids and few lived in a physical environment suitable for such aids.

### Gender differences

Previous research suggests women with disabilities experience a 'double disadvantage' [9-11]. In this current study, the female participants had less formal education, and a lower percentage of them were married at the time of the study. However, this may reflect the mean ages of males and females in the sample (i.e. older age range, older women) and the higher proportion of rural participants, rather than necessarily the contemporary interaction between gender and disability. In addition, findings indicate that men perceived their impairment to have greater impact on many life aspects, resulting in higher levels of distress. So while this paper does not dispute the disadvantages for women with disabilities in terms of objective and external circumstances such as income, employment, education, and so forth, it does raise issues as to gender differences in terms of subjective experiences of disability in Malaysia.

One of the key areas in which men indicated greater impact and/or distress than women was ability to participate in community activities and to move around the neighbourhood. It is possible that Malaysian men, particularly in rural settings, would be likely to engage in activities outside of the home more so than women, and hence a locomotive disability would be considered a greater interruption to their gendered role in the community. In particular for Muslim men, the dependence on others for mobility would limit their ability to attend the mosque for Friday prayers, partly because of difficulties kneeling (although they can pray while sitting in a wheelchair) but also because of questions of access. As one participant explained: "*I have to realise who I am ... I'm not like other people. I don't go to the mosque. It's difficult because I need to cross roads to go to the mosque. Cars can just pass and don't notice me at the roadside. So I think it's too dangerous*" (Malay, M).

In addition, men reported greater distress than women in relation to their ability to live independently, and in their overall satisfaction with life. Men also reported greater impact and distress on their ability to assist their family members. This may be related to conventional gender roles, with men feeling a duty to care for the family. As one woman stated in her interview, "*Men feel angry and frustrated because they can't contribute to the family ... they can't perform their duty to provide for their family. It is a blow to their morale*" (Malay, F).

However, it is possible that the gender differences found in this study are less a reflection of a greater subjective toll for men, but perhaps, more a reflection of differences in response style to such surveys. For example, previous findings suggest factors such as gender and lower socio-economic positions, as well as culture, anonymity and question type can impact on quality of data obtained, and the likelihood of participants acquiescing or giving socially desirable responses[23,24]. It is also possible that these findings reflect gender differences in tendency or ability to accept external circumstances. One of the female participants in the study reflected on the continued demands on women, regardless of their own health status, and stated: "*Women are very strong ... As a woman she can accept it (her impairment) even though she feels worried. In the case of men, if they get sick, it is terrible... For a woman, even though she's sick, she still has to cook. For men, they just lie down ... Women are tougher as they have to take care of their children, family and themselves*" (Malay, F)

Clearly, this paper provides preliminary data only, and further research is required to understand any possible differences among males and females with a disability in Malaysia. A limitation of this study is that the sample size is relatively small and heterogenous in regard to cause of mobility, and so generalization based on the findings is not possible. For instance, in the current study, males were more likely to have acquired their mobility impairment as a result of an accident, and hence, the gender differences found in this study may instead, be a reflection of this heterogeneity in the sample. Therefore, further research on possible gender differences should aim to limit the heterogeneity in their sample, or increase the sample size.

In addition, follow-up studies are required using other indicators of well-being, given the limitations of the EQ-5D in terms of its sensitivity[25] and that the PIPP is a new measure still undergoing the validation process for use in different cultural settings. Further research examining the different settings within Malaysia is also warranted. In this study, the majority of participants were from rural areas, recruited from health centres in the main town of the selected districts. For greater understanding of the experience of impaired mobility in rural Malaysia, more extensive ethnographic fieldwork is required in those areas. Future research would not only need to examine how individuals navigate the physical environment of rural Malaysia, but also their role within the community, as this study suggests the importance of participation in community-related activities. Clearly, further research is warranted on the impact of disability on perceptions of the private, public, and collective self, and on traditional gender roles.

Another limitation in the study is that almost 30 percent of the population were not Malay, and would have conducted their interviews in Mandarin, Cantonese or Tamil. Unfortunately, the questionnaire was not translated into these languages in as formal a process as it was for Malay, and hence poses potential bias in the results. Although this paper did not focus on residence or religion, this does not suggest any less relevance, and further research such as access to health care facilities and services, and meanings of disability in a religious context, is required.

This paper provides preliminary data in relation to health-related quality of life, and the impact and distress associated with impaired mobility in Malaysia. Despite limitations in the current study, it draws attention to the need to recognise the multifaceted nature of disability, and the diverse cultural contexts in which it can be experienced. In the current sample, issues of community involvement and participation were particularly significant. The findings also raised important questions regarding gender differences in the subjective experience of disability in Malaysia. Much research is still required for greater understanding of the social and cultural context of mobility limitations, poor health and aging, and of the heterogeneous ways in which these impact on individuals and families. Documentation of such information is essential to inform future policy initiatives and programmatic changes.

## Competing interests

The author(s) declare that they have no competing interests.

## Authors' contributions

LM, EB, and JP designed the study, and obtained the data with the help of ZO and RABR. RM conducted the statistical analyses and was responsible for the preparation of the article. Other authors also contributed to the preparation of the article and approved the final manuscript.
